# Consumer preferences for the development of new emulsion products based on vegetable and animal fats

**DOI:** 10.1371/journal.pone.0336602

**Published:** 2026-01-13

**Authors:** Małgorzata Kowalska, Magdalena Woźniak, Krzysztof Golec, Anna Zbikowska, Jerzy Szakiel, Paweł Turek

**Affiliations:** 1 Casimir Pulaski Radom University, Radom, Poland; 2 Medicofarma S.A., Warsaw, Poland; 3 Warsaw University of Life Sciences-SGGW (WULS-SGGW), Warsaw, Poland; 4 Krakow University of Economics, Krakow, Poland; Prince of Songkla University, THAILAND

## Abstract

The purpose of this paper was to determine consumer preferences for the acceptance of a new emulsion product containing interesterified fat based on mutton tallow and hemp oil. The survey concerned consumers’ reference to the new emulsion products as pharmaceutical, food, and cosmetic products. On the basis of the survey, it was concluded that the hypothetical emulsion products would gain consumer acceptance. Acquisition of this knowledge forms the basis for making decisions regarding the implementation of further experimental work, the outcome of which will be the evaluation of the properties of prototype products, including a comparative assessment of the sensory characteristics of prototypes and market products. More than half of the respondents answered that they would be interested in a new emulsion product based on interesterified fat. The most important factors indicated by the respondents that would lead them to purchase cosmetic or pharmaceutical emulsions were improvement in skin condition after application and the possibility of testing a free sample in advance. In the case of a food emulsion containing modified mutton tallow and hemp oil, on the other hand, the most important factors determining a potential purchase were the opportunity to try a free sample of the product in advance and attributes such as taste and texture that corresponded to the respondents.

## Introduction

Consumer research plays a key role in marketing strategy and product development, providing valuable information that helps businesses better understand their customers. This enables them to tailor their offerings to meet consumer expectations, which in turn increases the chances of market success [1,2].Consumer research provides information that can also inspire the development of new products or services [3]. Understanding what is missing from the market or what consumers’ unresolved problems are allows manufacturers to produce new products that do not yet exist or provides an opportunity to improve or simply modify them [4].

Regular surveys allow monitoring customer satisfaction levels, which is key to maintaining loyalty and long-term relationships. With feedback, it is possible to react quickly to potential problems and make appropriate adjustments to maintain sales continuity [5]. Consumer research can also help identify future trends, which is crucial in a rapidly changing market environment [6]. Anticipating what changes may occur in consumer preferences allows you to adjust your business strategy in advance.

The development of a new nutritional, cosmetic or pharmaceutical product is an activity that requires a combination of knowledge from various sciences, including food science, chemical science, marketing, and management [7]. Recently, more and more attention has been paid to the quality and safety of these products and its high value has been associated with this. The production of a safe and qualitatively good product is a multifaceted process that requires care at every stage of production. One of the important stages in the creation of a new product is the correct selection of raw materials and ingredients – more specifically, the concentration of substances that are responsible for specific functions in our bodies. In recent years, there has been a noticeable increase in interest in products based on natural ingredients [8–10]. Consumers are increasingly looking for alternatives that are compatible with a healthy and rational diet and, when it comes to cosmetic products, compatible with and have affinity for the skin.

An unusual alternative for use as a raw material in cosmetic, pharmaceutical, or food emulsions may be mutton tallow due to its known moisturising, nourishing, or protective properties. It shows biocompatibility with human skin and contains fat-soluble vitamins A, D, E, and K, which play a key role in skin regeneration and maintenance [11].

Hemp oil, extracted from the seeds of the hemp plant, in turn, is a valued ingredient in cosmetic formulations, nutritional emulsions, and also in pharmaceutical products. Interest in this oil is dictated by its characteristic fatty acid composition, namely its high content of valuable unsaturated fatty acids (80–90%), including omega 3 and omega 6. Unrefined, it is also rich in numerous B vitamins, vitamin K, as well as tocopherols (vitamin E), carotenoids (vitamin A), polyphenols, sterols or chlorophylls [12].

Given these properties of the two ingredients, a consumer survey was carried out in the study to obtain consumer opinion on whether it is reasonable to produce a new fat that could be an ingredient in future food, cosmetic, or pharmaceutical products. The study asked consumers whether they would be interested in an emulsion product based on an interestrified fat derived from the two natural ingredients hemp oil and sheep tallow [13,14]. The purpose of this study was to obtain an opinion on whether such a modification was justified and whether the proposed new product would be of interest to consumers participating in the study.

## Material, methods

### Survey

The production of new products based on natural raw materials does not always meet consumer expectations, which is why producers or technologists introduce modifications to obtain a product as close as possible to consumer preferences. Therefore, one of the tasks of successfully gaining acceptance for a new product is to carry out a consumer survey in this area and, once the results have been obtained, to proceed with the creation of a new product that is attractive from a nutritional point of view.

The survey studies conducted do not fall into the category of clinical trials and do not require approval from ethics committees under the provisions of the Declaration of Helsinki. Nevertheless, all survey procedures were carried out in accordance with the Code of Good Practices in Universities developed by the Polish Rectors Foundation and adopted by the Plenary Assembly of the Conference of Rectors of Academic Schools in Poland (CRASP) on April 26, 2007. Furthermore, the procedures complied with the ethical standards in force at Casimir Pulaski Radom University, as set out in ORDER R-77/2013 of the Rector dated October 31, 2013, which introduced the Code of Ethics for Academic Teachers. The survey was entirely anonymous and participation was voluntary—respondents could withdraw at any stage without providing a reason.

Information about the voluntary nature of participation and the expression of informed consent to participate in the study was included in the introduction to the survey form. The study did not include participants under 18 years of age.

In this study, a questionnaire survey was conducted to gain knowledge about the acceptance of emulsion products made from enzyme-modified fat mixtures of sheep tallow and hemp oil. At the same time, the study sought to identify factors that could influence consumers’ purchase decisions for the proposed products. The survey introduction provided participants with key information on the source and properties of sheep tallow and hempseed oil. This included details on their potential application in cosmetics and food products, such as their use as a substitute for synthetic components.

The survey was carried out using the CAWI (*computer-assisted web interview*) method. The survey questionnaire consisted of 8 closed questions, single or multiple choice. The survey was conducted through the Ankieteo.pl website. The research sample was random, and the sampling frame was SWpanel.pl, a panel run by the SW Research agency.

## Results and discussion

Within the framework of the research 1000 questionnaires were conducted. Participation in the research was voluntary and anonymous. The structure of the respondents in terms of gender, age, education, and place of residence was presented in Table 1.

**Table 1:** General structure of respondents (N = 1000).

Specification	Percentage (%)
Gender	
woman	71.0
man	29.0
Age	
18-25 years old	36.4
26 - 45 years	41.8
46 - 60 years	15.8
over 61 years	6.0
Education	
Primary education (ISCED 1–2)	3.9
Lower secondary education (ISCED 2)	8.2
Basic vocational (ISCED 3)	12.0
Upper secondary education (EQF Level 4)	45.3
Higher education (EQF Level 6+)	30.6
Place of residence	
village	34.4
a city with up to 50,000 inhabitants	21.1
a city with 51,000–150,000 inhabitants,	15.3
a city with 151,000–500,000 inhabitants	14.9
a city with more than 500,000 inhabitants	14.3

**Fig 1:**
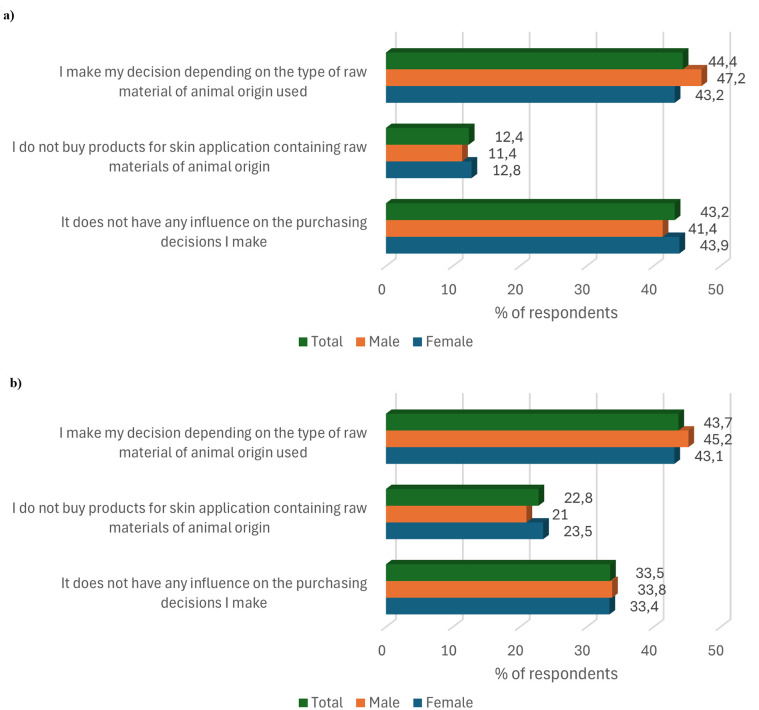
Relationship between the purchase decision of products a) food and b) for skin application in the context of the presence of raw materials of animal origin in them, according to the sex of the respondents.

In the first two questions, respondents were asked about their preferences regarding the purchase of food products and those intended for use on the skin (cosmetics, medicinal ointments) containing raw materials of animal origin. The results are presented in Fig 1. Of 1000 respondents, 43.2% declared that ingredients of animal origin in food products do not have any influence on their purchasing decisions. In contrast, one in three respondents indicated that purchasing a product for skin application that contains an ingredient of animal origin is not a problem for them. A similar number of respondents indicated that their purchasing decisions for both food and skin care products depend on the type of animal raw material used (44.4 and 43.7% respectively). On the other hand, 12.4% of the respondents declared that ingredients of animal origin disqualify the purchase of a food product, and almost twice as many (22.8%) indicated that they do not buy products intended for use on the skin containing animal ingredients.

In terms of purchasing decisions regarding food products containing raw materials of animal origin, male respondents depended on the type of raw material used to a greater extent (difference of 4.0 percentage points) than female respondents in their purchasing decisions. In contrast, more women than men indicated that they do not buy this type of product (difference 1.4 percentage points) and that this factor does not influence their purchasing decisions (difference 2.5 percentage points).

Slightly smaller differences were observed between the responses of both genders for the question about products intended for use on the skin than for foods containing ingredients of animal origin. Respondents of both genders declared to a similar extent that the presence of animal ingredients in products for skin application does not influence the decision to buy them. One in three respondents chose this answer, with a gender difference of 0.4 percentage points in favour of male respondents. Slightly more female than male (difference 2.5 percentage points) declared that they do not buy skin care products containing raw materials of animal origin. A higher number of male respondents than female respondents (difference 2.1 percentage points) indicated that their purchase decision for these products is influenced by the animal raw material used.

The animal-based food products did not depend on the gender of the respondents (chi2(2, N = 1000) = 1.399, p = .50, V = .04), their age (chi2(6, N = 1000) = 5.777, p = .50, V = .05), education (chi2(8, N = 1000) = 12.971, p = .11, V = .08) or place of residence (chi2(8, N = 1000) = 8.145, p = .50, V = .06).). Based on the results of the statistical analysis on consumer preferences in the context of purchasing products for skin application containing raw materials of animal origin, it was found that they did not depend on the gender of the respondents (*chi2*(2, *N* = 1000) = 0.772, *p = *.68, V = .03), their age (*chi2*(6, *N = 1000)* = 3.797, *p = .*70, V = .04), education (*chi2*(8, *N = 1000)* = 13.067, *p = .*11, V = .08), or place of residence (*chi2*(8, *N = 1000)* = 7.544, *p = .*48, V = .06).

**Fig 2:**
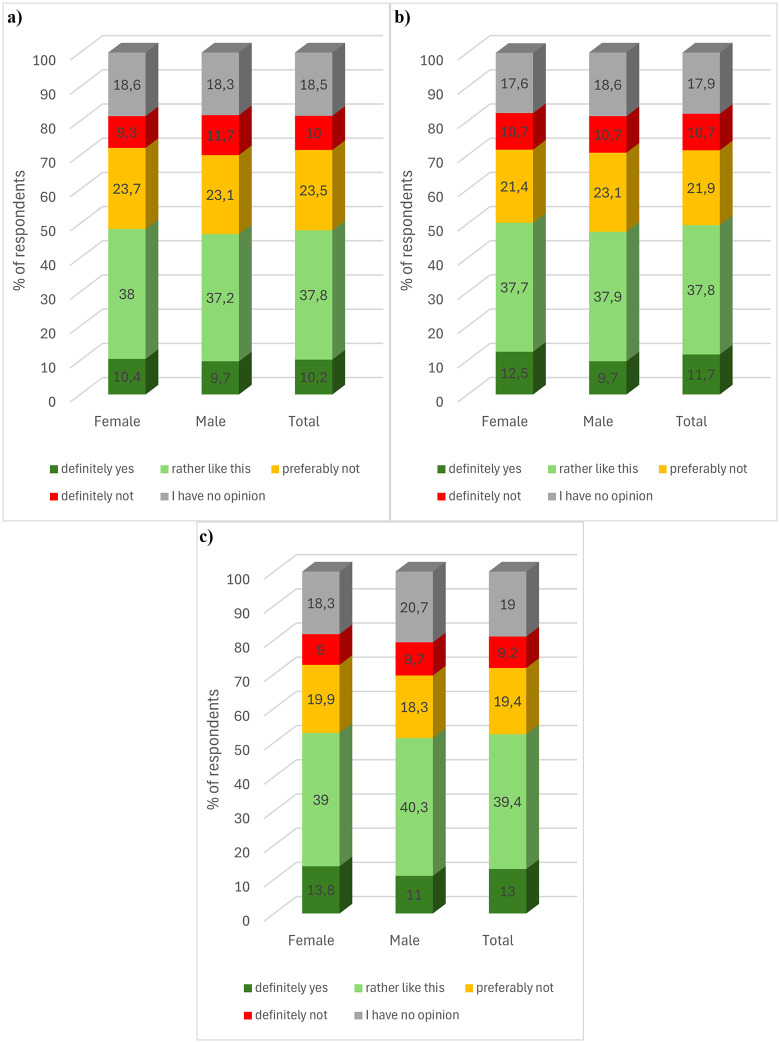
Consumer preferences in terms of willingness to pay a higher price for a) food, b) cosmetics, and c) pharmaceutical product containing interesterified fat, by gender. **(a)** Food product. **(b)** Cosmetic product.

In the next question, respondents were familiarised with the terminology associated with the process of interesterification to introduce the topic of the question. Respondents were asked whether they would be willing to pay a higher price for products based on interesterified fat. The results showed that about half of the respondents would be willing to pay more for such a product (Fig 2). The sum of the total number of yes and definitely yes answers for food, cosmetics and pharmaceutical products was 48.0, 49.5 and 52.4% respectively. In contrast, almost one in three respondents declared that they would not pay a higher price for a product containing interesterified fat. The sum of the total of the rather than and definitely not responses for food, cosmetics and pharmaceutical products was 33.5, 32.6 and 28.6% respectively. The results show that respondents were most willing to pay a higher price for a pharmaceutical product containing enzymatically modified fat, and least willing to pay a higher price for a food product. About one in five respondents had no opinion on this issue.

Statistical analysis showed that the’ gender of the respondents did not statistically significantly differentiate their preference for willingness to pay an increased price for a food product (chi2(4, N = 1000) = 1.071, p = .90, V = .03), a cosmetic product (chi2(4, N = 1000) = 1.995, p = .74, V = .04), as well as pharmaceutical (chi2(4, N = 1000) = 2.102, p = .71, V = .05) containing interesterified fat. Taking into account the age of the respondents, it was found that it did not statistically significantly differentiate the willingness to pay an increased price for a food product (chi2(12, N = 1000) = 13.687, p = .32, V = .07) and a pharmaceutical (chi2(12, N = 1000) = 15.768, p = .20, V = .07).). However, a statistically significant relationship was found in a non-monotonic sense between age and willingness to pay a higher price for a cosmetic product (chi2(12, N = 1000) = 21.556, p = .04, V = .08). However, on the basis of the value of Cramer’s V coefficient, the strength of the relationship was found to be very weak. The proportion of individual responses to the question analysed was not statistically significant depending on the respondents’ place of residence (food product (*chi2*(16, *N* = 1000) = 20.879, *p = *.18, V = .07), cosmetic (*chi2*(16, *N = 1000)* = 23.497, *p = .*10, V = .08), pharmaceutical (*chi2*(16, *N = *1000) = 18.632, *p = .*29, V = .07)).

**Fig 3:**
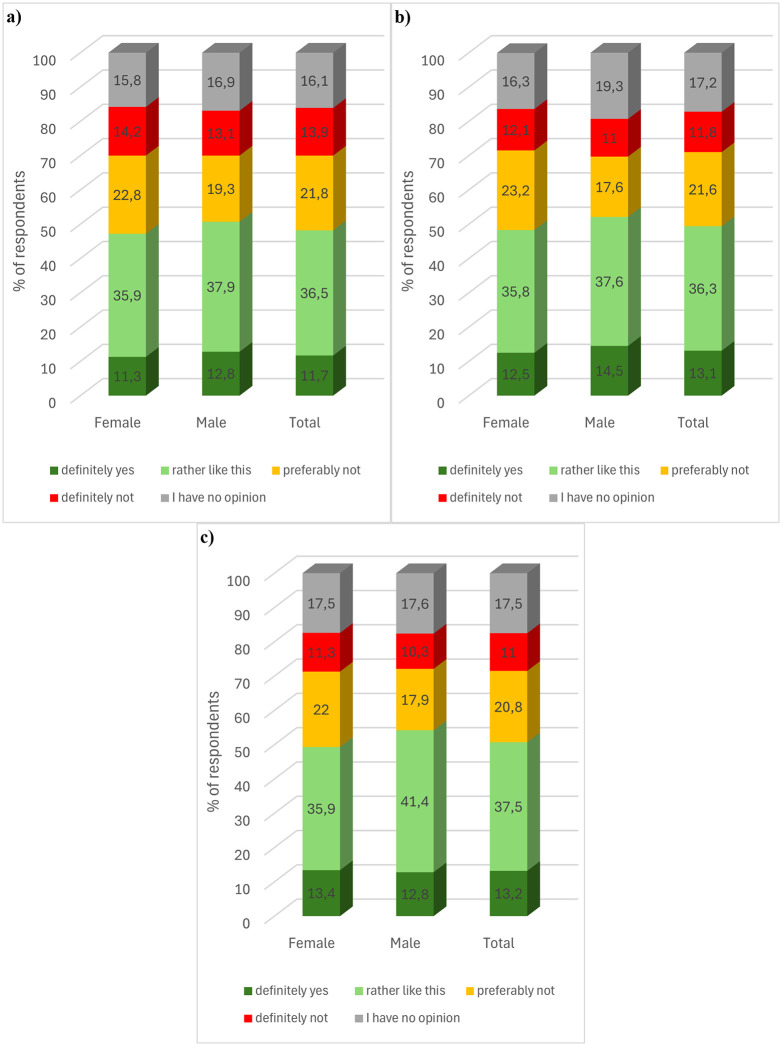
Consumer preferences for purchasing a) food, b) cosmetic, and c) pharmaceutical product containing mutton tallow. **(a)** Food product (b) Cosmetic product.

In the survey conducted, respondents were also asked to refer to the purchase of mutton tallow-based products. Approximately half of the respondents would buy such products (Fig 3). The sum of the *rather yes* and *definitely yes* responses was the highest (50.7%) for pharmaceutical products and the lowest for food products (48.2%). On the other hand, 49.4% of the respondents stated that they would buy a cosmetic product containing mutton tallow. Approximately 1/3 of the respondents declared to have a negative opinion on the purchase of products containing this animal fat. The sum of *preferably not* and *definitely not* responses for food, cosmetic and pharmaceutical products was 35.7, 33.4, and 31.8% respectively. Based on the results obtained it can be concluded that pharmaceutical products containing mutton tallow were characterised by the highest acceptance among the respondents, while food products were characterised by the lowest. About one in six respondents did not have an opinion on this issue.

Statistical analysis showed that consumer preferences for the purchase of food products (chi2(4, N = 1000) = 2.042, p = .73, V = .04), cosmetic products (chi2(4, N = 1000) = 5.495, p = .24, V = .07) and pharmaceutical products (chi2(4, N = 1000) = 3.645, p = .46, V = .06) containing mutton tallow were not statistically significant depending on the’ gender of the respondents. However, the age and level of education of the respondents statistically significantly differentiated the answers to the question asked. In general, younger respondents were less convinced that they would decide to buy food, cosmetic and pharmaceutical products containing mutton tallow (Kendall’s tau-c = −.054, approximate T = −2.196, *p* = .028; Kendall’s tau-c = −.090, approximate T = −3.544, *p* = .000; Kendall’s tau-c = −.069, approximate T = −2.764, p = .006). The correlation between the variables for all types of products analysed was found to be statistically significant, although with zero strength of association. The preference for purchasing food products containing mutton tallow did not depend on the ‘ level of education of the respondents (chi2(16, N = 1000) = 18.204, p =.731, V =.07). In contrast, respondents with lower levels of education were less confident that they would choose to purchase cosmetic and pharmaceutical products containing mutton tallow (Kendall’s tau-b = -.068, approximate T = -2.554, p = 0.011; Kendall’s tau-b = -.099, approximate T = -3.663, p =.000).). The correlation coefficient, although statistically significant, had practically zero strength of association. The’ place of residence of the respondents did not statistically significantly differentiate the responses to the question asked in the case of food products (chi2(16, N = 1000) = 16.662, p = .41, V = .07), cosmetics (chi2(16, N = 1000) = 16.189, p = .44, V = .06) and pharmaceuticals (chi2(16, N = 1000) = 20.026, p = .22, V = .07)).

**Fig 4:**
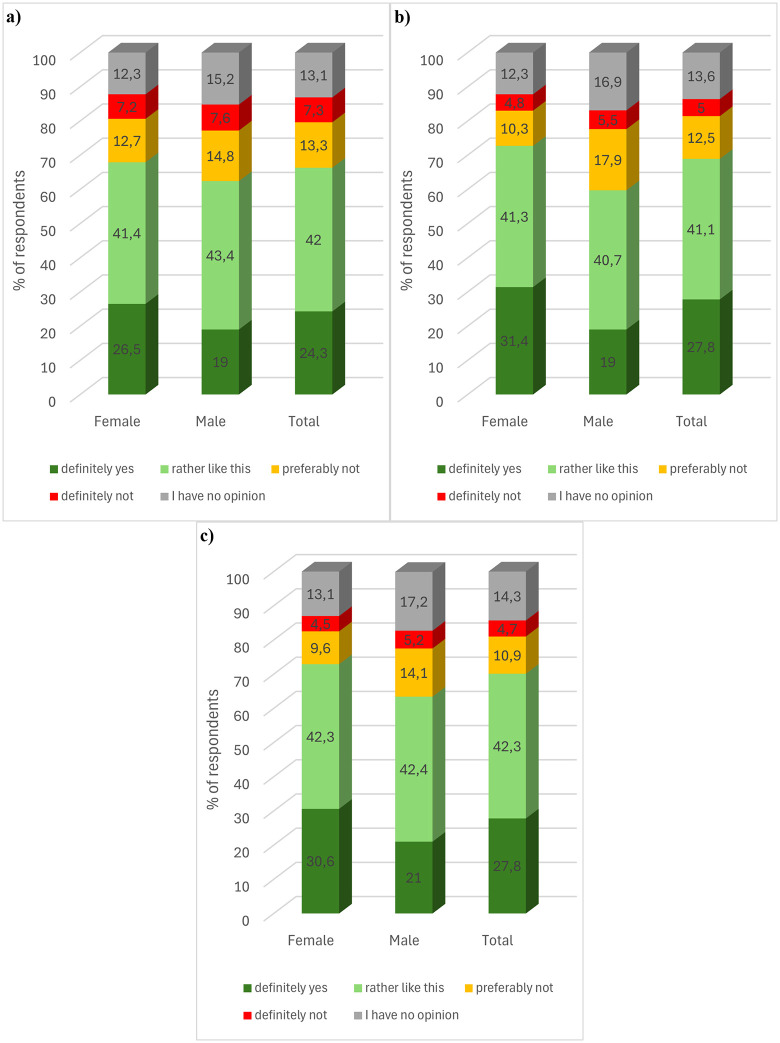
Consumer preferences for purchasing a) food, b) cosmetic, and c) pharmaceutical product containing hemp seed oil. **(a)** Food product **(b)** Cosmetic product.

In the next question, respondents were asked to refer to the purchase of products containing hemp seed oil. The question was preceded by brief information on the properties of hemp seed oil. In general, respondents showed greater acceptance for food, cosmetic, and pharmaceutical products containing hemp seed oil than for those containing mutton tallow. Approximately two-thirds of the respondents would purchase all three proposed types of products containing hemp seed oil (Fig 4). The sum of responses that indicated a positive attitude towards the proposed products, i.e., rather yes and definitely yes, was the highest for pharmaceutical products and reached 70.1%. A slightly lower sum of these responses was recorded for cosmetic products (68.9%) and food products (66.3%). About one in five respondents had a negative attitude towards the proposed food products by choosing preferably not or definitely not answers (total responses 20.6%). In the case of cosmetics and pharmaceuticals, the sum of responses indicating unwillingness to buy such products was 17.5 and 15.6% respectively. About one in seven respondents had no opinion.

Statistical analysis showed that female respondents, compared to male respondents, were more confident that they would choose to purchase a cosmetic product (*chi*^*2*^(4, *N* = 1000) = 24.434, *p* = .00, V = .16) and a pharmaceutical product (*chi*^*2*^(4, *N* = 1000) = 13.183, *p* = .01, V = .11) containing hemp seed oil. The relationship between the variables was statistically significant, although the strength of the relationship was insignificant. However, in the case of purchasing food products containing the indicated oil, no statistically significant differences were found (chi2(4, N = 1000) = 7.118, p = .13, V = .08) in the context of the’ gender of the respondents. Statistically significant non-monotonic relationships with insignificant strength of association were found between age and purchase preferences for food products (chi2(12, N = 1000) = 22.765, p = .03, V = .09) and pharmaceuticals (chi2(12, N = 1000) = 21.875, p = .04, V = .08).). However, this variable did not affect the’ preferences of the respondents for cosmetic products (chi2(12, N = 1000) = 16.324, p = .18, V = .07). Moreover, respondents with lower levels of education were found to be less convinced about the purchase of food products (Kendall’s tau-b = −.080, approximate T = −2.921, p = .003), cosmetic products (Kendall’s tau-b = −.099, approximate T = −3.712, p = .000), as well as pharmaceutical products (Kendall’s tau-b = −.089, approximate T = −3.237, p = .001) that would contain hemp Although statistically significant, the correlation coefficient for these variables had practically zero strength of association. The residence’ of the respondents did not statistically significantly differentiate the answers to the question asked in the case of cosmetic products (chi2(16, N = 1000) = 21.577, p = .16, V = .07) and pharmaceutical products (chi2(16, N = 1000) = 17.203, p = .37, V = .07). In the case of preferences regarding the purchase of food products containing hemp seed oil, it was found that respondents living in less urbanised towns were slightly less convinced that they would decide to buy it (Kendall’s tau-b = −.056, approximate T = −2.0411, p = 0.041).). The correlation coefficient was statistically significant, but had virtually zero strength of association.

**Fig 5:**
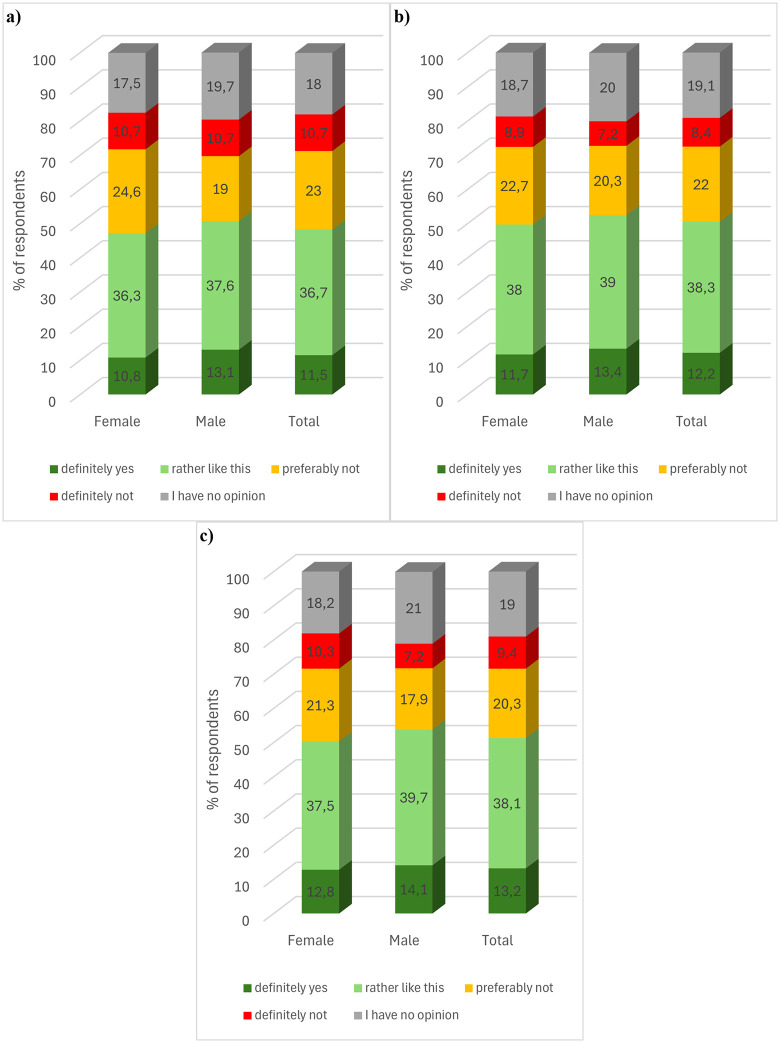
Consumer preferences to purchase a) food, b) cosmetic, and c) pharmaceutical product containing as one of its ingredients an enzyme-interesterified blend of mutton tallow and hemp seed oil. **(a)** Food product **(b)** Cosmetic product.

Respondents were also asked whether they would purchase products containing enzyme-interesterified mutton tallow with hemp seed oil. Approximately half of the respondents declared that they would decide to make such a purchase (Fig 5). A pharmaceutical product based on the mentioned fat was characterised by the highest acceptance level. The sum of the rather yes and definitely yes answers was 51.3%. Slightly less acceptance was found for cosmetic and food products containing modified fat (sum of the answers 50.5 and 51.3% respectively). The sum of the answers indicating a negative attitude towards the proposed products, i.e., preferably not and definitely not, was the highest for food products (33.7%), and the lowest for pharmaceuticals (29.7%). The results obtained are consistent with the information provided by Gutkowska et al. (2009). The authors indicated that consumer innovativeness towards new food products is at a relatively low level. The authors mentioned that it may result, among others, from the fact that purchase decisions concerning food result from habits and hedonistic expectations of consumers. In the case of cosmetics, the sum of responses indicating a negative attitude was 30.4%. About one in five respondents had no opinion.

Consumer preferences for purchasing food (chi2(4, N = 1000) = 4.111, p = .39, V = .06), cosmetic (chi2(4, N = 1000) = 2.326, p = .68, V = .05) and pharmaceutical products (chi2(4, N = 1000) = 5.309, p = .26, V = .07) containing an enzymatically interesterified blend of mutton tallow and hemp seed oil were not statistically significantly dependent on the sex of the respondents. The younger respondents were less confident that they would choose to purchase food products (Kendall’s tau-b = −.057, approximate T = −2.273, p = .023) and pharmaceutical products (Kendall’s tau-b = −.080, approximate T = −3.192, p = .001) that contain the proposed modified fat blend. There was a statistically significant but non-monotonic relationship between age and preference to purchase a cosmetic product with the indicated ingredient (*chi*^*2*^(12, *N* = 1000) = 25.700, *p* = .01, V = .09). The level of education did not statistically significantly differentiate purchase preferences for food (*chi*^*2*^(16, *N* = 1000) = 12.950, *p* = .68, V = .06) and cosmetic products (*chi*^*2*^(16, *N* = 1000) = 8.920, *p* = .92, V = .05) containing the proposed modified fat. However, respondents with a lower level of education showed less conviction to purchase pharmaceutical products containing the indicated fat (Kendall’s tau-b = −.070, approximate T = −2.625, p = .009). The respondents’ place of residence did not statistically significantly differentiate the answers to the question in the case of all analysed products (food (*chi*^*2*^(16, *N* = 1000) = 17.959, *p* = .33, V = .07), cosmetic (*chi*^*2*^(16, *N* = 1000) = 14.342, *p* = .57, V = .06), pharmaceutical (*chi*^*2*^(16, *N* = 1000) = 18.399, *p* = .30, V = .07)).

The next question concerned the respondents’ evaluation of a skin care product based on an interesterified fat. The respondents were asked to indicate the most important features of the product mentioned that would contribute to their decision to purchase it (Table 2). The improvement in skin condition attribute after application was identified as the main purchase factor for the proposed product by 51.4% of women, while it was less important for men (32.4%). The opportunity to test a free sample beforehand, price, sensory experience and the opinion of others were also important factors chosen by respondents. All these features were indicated by significantly more female respondents than male respondents. Gender differences ranged from 5.9 to 10.1 percentage points. In the case of product originality, on the other hand, significantly more men (22.8%) than women (17.7%) identified it as a determining factor for the purchase of the proposed product. Of the purchase determinants proposed in the survey, the least influential were the type of packaging and advertising, declared by 11.0% and 7.9% of the total respondents respectively. 6.3% of the respondents declared that they would not buy the proposed product and 8.9% declared that they had no opinion.

**Table 2:** Determinants of purchase of a product intended for use on the skin (cream, lotion) containing interesterified fat, indicated by women and men.

Factor	% Women	% Men	% Total
Improved skin condition after application (adequate hydration, reduced transepidermal water loss)	51.4	32.8	46.0
Opportunity to test a free sample beforehand	43.1	37.2	41.4
Price	40.4	30.3	37.5
Adequate sensory perception on the skin during and after application (such as consistency, spreading over the skin, absorption, odour)	29.2	23.1	27.4
The opinion of others	28.3	22.1	26.5
Originality of the product	17.7	22.8	19.2
The type of product packaging that suits me	11.4	10.0	11.0
I have no opinion	6.8	14.1	8.9
Advertising (social media, press, television)	8.6	6.2	7.9
I would not buy such a product	5.4	8.6	6.3
Other	0.3	0.3	0.3

Statistical analysis showed that the factors that were statistically significantly determined by the gender of respondents were: improvement of skin condition after application, opinion of others, price and no opinion. Younger respondents statistically significantly more often indicated that factors that could contribute to the purchase of the proposed product were: improvement of skin condition after application, opinion of others, and price. Older respondents were statistically significantly more likely to indicate that the possibility of testing a free sample beforehand would be the deciding factor for a purchase. Respondents with a higher level of education were statistically significantly more likely than others to indicate that the factors that could contribute to the purchase of the proposed product are: improvement in skin condition after application, sensory experience and the ability to test a free sample. The only factor influenced by the respondents’ place of residence was the sensory experience after product application. This factor was selected significantly more often by people living in more urban locations.

The next question in the survey was related to the preferences of consumers for a food product (e.g., salad dressing) containing interesterified fat. Respondents were asked to indicate the factors that could contribute to their purchase of such a product. The answers obtained are presented in Table 3. Slightly more female than male respond (30.8% and 28.6%, respectively) indicated the price as a factor determining the purchase of the proposed product. However, among the male respondents, it was the price that was indicated as the main motivator to purchase the proposed product. Women, on the other hand, most often indicated the possibility to try a free sample of the product beforehand (34.9%). The difference between women’s and men’s preference for this factor was 7 percentage points. Sensory attributes, the health-promoting properties of the product and the opinion of others were also important factors chosen by respondents for the mentioned product. The influence of these factors was declared by more female respondents (33.5%, 33.1% and 25.2%) than male respondents (27.2%, 22.4% and 21.0%). On the other hand, more male respondents (26.2 and 21.4%) than female respondents (22.0% and 16.1%) indicated functional features and product originality as determinants. Of the factors mentioned in the survey that could influence the decision to buy a product, the least influential was advertising, declared by 5.8% of women and 9.0% of men respectively. 11.1% of the total number of respondents declared that they would not buy the proposed product, the same number of respondents indicated that they had no opinion on this matter.

**Table 3:** Determinants of purchase of a food product containing interesterified fat, indicated by women and men.

Factor	% Women	% Men	% Total
Opportunity to try a free sample of the product before the trial.	34.9	27.9	32.9
Sensory attributes (taste, texture, etc.)	33.5	27.2	31.7
Price	30.8	28.6	30.2
The health promoting properties of the product	33.1	22.4	30.0
The opinion of others	25.2	21.0	24.0
Functional characteristics (convenience, durability, accessibility, type of packaging)	22.0	26.2	23.2
Originality of the product	16.1	21.4	17.6
I would not buy such a product	12.0	9.7	11.1
I have no opinion	11.4	10.3	11.1
Advertising (social media, press, television)	5.8	9.0	6.7
Other	0.3	0.3	0.1

The proportion of the following determinants of purchase of the proposed food product was statistically significantly influenced by the gender of the respondents: the possibility to try a free sample beforehand, the health-promoting properties, and the originality of the product. The only factor that the younger respondents indicated statistically significantly more often than the older respondents was advertising. Respondents with basic vocational education statistically significantly less often than others indicated the following determinants: sensory attributes, functional attributes, and pro-health properties of the product. More educated respondents indicated, to a statistically significant greater extent than others, that the possibility of trying a free sample of the product beforehand was an important factor. No opinion was statistically significantly more apparent among respondents with lower education level. Taking into account the place of residence of the respondents, no statistically significant relationship was found between this variable and factors that could contribute to the purchase of the proposed product.

## Conclusions

The survey showed that more than half of the respondents would be interested in a new emulsion product, intended for food use and for skin application, based on an interesterified fat containing mutton tallow and hemp seed oil. Among the types of target products proposed, the highest level of acceptance was for potential pharmaceutical products and the lowest for food products. The respondents indicated that the most important determinants that would lead them to purchase an emulsion product for skin application containing modified mutton tallow and hemp seed oil would be the improvement in skin condition after application and the opportunity to test a free sample in advance. If the proposed product was intended for food use, the most important factors that would induce respondents to purchase it were the opportunity to try a free sample of the product in advance and attributes such as taste and texture that respondents liked.

The results obtained can be a valuable source of information for technologists or manufacturers and can also guide the development of new formulations of dispersion systems as model systems for cosmetic or pharmaceutical food products.

## References

[1] Luchs MG, Swan KS, Creusen MEH. Perspective: A review of marketing research on product design with directions for future research. J of Product Innov Manag. 2015;33(3):320–41. doi: 10.1111/jpim.12276

[2] Marion TJ, Friar JH, Simpson TW. New product development practices and early‐stage firms: two in‐depth case studies. J of Product Innov Manag. 2012;29(4):639–54. doi: 10.1111/j.1540-5885.2012.00930.x

[3] Steffen A. Exploring the benefits of employing market insights and consumer trends in food product innovation: A case study from Germany. In: Cavicchi A, Santini C, editors. Case Studies in the Traditional Food Sector. Woodhead Publishing; 2018. p. 209–37.

[4] Siegrist M, Hartmann C. Consumer responses to plant-based and animal-based emulsified products: A comparative study. Food Quality and Preference. 2022;99:104558.

[5] Endrizzi I, Gasperi F. Consumer acceptance of plant-based and hybrid emulsified products: A systematic review. Food Quality and Preference. 2024;110:104678.

[6] Guiné RPF, Florença SG, Barroca MJ, Anjos O. The link between the consumer and the innovations in food product development. Foods. 2020;9(9):1317. doi: 10.3390/foods9091317 32962007 PMC7554954

[7] Aschemann-Witzel J, Varela P. Consumer perception of plant-based proteins. Trends in Food Science & Technology. 2019;91:196–204.

[8] Cliceri D, Spinelli S. Consumer acceptance and perceived sensory characteristics of commercial vegan mayonnaise. Foods. 2024;13(9):1542. 40361623 10.3390/foods14091542 PMC12071783

[9] Hoek AC, Luning PA. Consumer acceptance of blending plant-based ingredients into traditional meat-based foods. Food Quality and Preference. 2019;79:103754.

[10] Punter PH, de Jong J. Sensory and emotional responses to plant-based and animal-based emulsions. Food Quality and Preference. 2021;89:104120.

[11] Kowalska M, Woźniak M. Proposal of new emulsion systems containing hydroxypropylmethylcellulose as a viscosity modifier and diacylglycerols from mutton tallow and hemp seed oil. Applied Sciences. 2023;13(18):10289. doi: 10.3390/app131810289

[12] Pei L, Luo Y, Gu X, Wang J. Formation, Stability and Properties of Hemp Seed Oil Emulsions for Application in the Cosmetics Industry. Tenside Surfactants Detergents. 2020;57(6):451–9. doi: 10.3139/113.110712

[13] Chen J. Enzymatic interesterification of hybrid fats. Journal of Agricultural and Food Chemistry. 2022;70(12):3781–390.

[14] Kowalska M, Woźniak M, Turek P, Żbikowska A. New fat bases in model emulsion systems in physicochemical and consumer evaluation. Applied Sciences. 2024;14(9):3553. doi: 10.3390/app14093553

